# Sex Differences in Biophysical Signatures across Molecularly Defined Medial Amygdala Neuronal Subpopulations

**DOI:** 10.1523/ENEURO.0035-20.2020

**Published:** 2020-07-02

**Authors:** Heidi Y. Matos, David Hernandez-Pineda, Claire M. Charpentier, Allison Rusk, Joshua G. Corbin, Kevin S. Jones

**Affiliations:** 1 Center for Neuroscience Research, Children’s National Medical Center, Washington, DC 20010; 2 Department of Pharmacology, University of Michigan, Ann Arbor, MI 48109; 3 IBS Graduate Program, The George Washington University School of Medicine, Washington, DC 20052

**Keywords:** intrinsic properties, ion channels, medial amygdala, sexual dimorphism, spike adaptation, spike frequency

## Abstract

The medial amygdala (MeA) is essential for processing innate social and non-social behaviors, such as territorial aggression and mating, which display in a sex-specific manner. While sex differences in cell numbers and neuronal morphology in the MeA are well established, if and how these differences extend to the biophysical level remain unknown. Our previous studies revealed that expression of the transcription factors, Dbx1 and Foxp2, during embryogenesis defines separate progenitor pools destined to generate different subclasses of MEA inhibitory output neurons. We have also previously shown that *Dbx1*-lineage and *Foxp2*-lineage neurons display different responses to innate olfactory cues and in a sex-specific manner. To examine whether these neurons also possess sex-specific biophysical signatures, we conducted a multidimensional analysis of the intrinsic electrophysiological profiles of these transcription factor defined neurons in the male and female MeA. We observed striking differences in the action potential (AP) spiking patterns across lineages, and across sex within each lineage, properties known to be modified by different voltage-gated ion channels. To identify the potential mechanism underlying the observed lineage-specific and sex-specific differences in spiking adaptation, we conducted a phase plot analysis to narrow down putative ion channel candidates. Of these candidates, we found a subset expressed in a lineage-biased and/or sex-biased manner. Thus, our results uncover neuronal subpopulation and sex differences in the biophysical signatures of developmentally defined MeA output neurons, providing a potential physiological substrate for how the male and female MeA may process social and non-social cues that trigger innate behavioral responses.

## Significance Statement

The amygdala is a major brain center for processing environmental cues for social, emotional and survival behaviors. Furthermore, the amygdala is one of a handful of sexually dimorphic regions of the brain. Focusing on the medial subnucleus of the amygdala (MeA), which regulates innate social and non-social behaviors, here we studied neuronal subpopulation and sex differences in the intrinsic biophysical properties of two developmentally and molecularly identifiable subpopulations of MeA output neurons. We find dramatic lineage and sex differences in a variety of intrinsic biophysical properties and provide insight into potential molecular mechanisms underlying these differences. These sex-specific neural substrates may help us understand how the amygdala processes innate social and non-social behaviors differently across sex.

## Introduction

The medial amygdala (MeA) is a major brain nucleus for distinguishing olfactory cues that drive innate behaviors such as mating, territorial defense, predator avoidance and maternal and paternal care (McCarthy and Arnold, 2011; Sokolowski and Corbin, 2012; Bergan et al., 2014; Yang and Shah, 2014; Li and Dulac, 2018). In both females and males, the MeA responds to both sex-specific (e.g., opposite sex odors) and non-sex-specific (e.g., predator odor) cues (Choi et al., 2005; Wu et al., 2009; Root et al., 2014; Carvalho et al., 2015; Unger et al., 2015; Bayless and Shah, 2016; Lischinsky et al., 2017; Yao et al., 2017). In addition, MeA neurons in females and males display differences in a number of anatomic, morphologic, and molecular characteristics, including cell number, dendritic complexity, and gene expression patterns (Cooke et al., 1999; Cooke and Woolley, 2005; Johnson et al., 2008; Morris et al., 2008; Wu et al., 2009, 2017; Unger et al., 2015; Chen et al., 2019; Gegenhuber and Tollkuhn, 2019). However, how these properties converge to perform complex computations in the MeA in both sexes remain unknown. One critical missing piece to this understanding is putative differences in intrinsic biophysical properties of neurons, such as action potential (AP) firing patterns, which define how a neuron transmits information to downstream targets.

The MeA is comprised of a large variety of neurons, which include diverse subclasses of interneurons and both inhibitory and excitatory projection neurons (Bian, 2013; Keshavarzi et al., 2014; Hashikawa et al., 2018; Canteras et al., 2019; Chen et al., 2019). Although a full cataloging of MeA neuronal diversity remains incomplete, we have previously leveraged embryonic transcription factor expression patterns as a means to classify adult MeA neuronal diversity (Hirata et al., 2009; Carney et al., 2010; Lischinsky et al., 2017). We previously revealed that the embryonically expressed transcription factors, Dbx1 and Foxp2, define different embryonic progenitor pools that are destined to generate two molecularly and electrophysiologically distinct subclasses of mature MeA inhibitory output neurons. We have further shown that MeA *Dbx1-*lineage and *Foxp2*-lineage neurons respond in a sex and lineage-specific manner to aggressive, defensive, or mating cues (Lischinsky et al., 2017). Understanding both sex and lineage-specific differences in neuronal biophysical properties is a critical step to ultimately understand how different neuronal populations in the male and female MeA processes sensory information for appropriate behavioral outputs.

In this study, we conducted multidimensional analyses of the biophysical signatures and intrinsic electrophysiological profiles of *Dbx1-*lineage and *Foxp*2-lineage neuronal subclasses in the MeA in both adult females and males. We observed striking differences in spiking patterns across *Dbx1-*lineage and *Foxp2*-lineages, and also across sex within each lineage. We further uncovered sex and lineage differences in a host of intrinsic biophysical properties, including capacitance and spike-frequency adaptation, the latter of which is known to be modified by specific voltage-gated ion channels. To identify a potential mechanism underlying our observed sex and lineage differences in spike adaptation, we conducted a novel approach to the phase plot analysis of the AP waveform to narrow down putative ion channel candidates. Of these candidates, a subset was expressed in either a sex-biased or lineage-biased manner. Thus, by uncovering sex and lineage differences in intrinsic biophysical profiles of molecularly identifiable MeA output neurons, our results provide a potential physiological substrate for how subclasses of neurons in the female and male MeA may process social and non-social cues.

## Materials and Methods

### Animals

Mice were housed in the temperature and light-controlled (12/12 h light/dark cycle) animal care facilities at the University of Michigan and Children’s National Medical Center and given food and water *ad libitum*. All animal procedures were approved by the University of Michigan and Children’s National Medical Center’s Institutional Animal Care and Use Committees (IACUC) and conformed to NIH Guidelines for animal use. Mice used were *Dbx1^cre^*+/− (kindly provided by A. Peirani, Institut Jacques Monod, Paris; Pierani et al., 2001) and *Foxp2^cre^*+/− (kindly provided by R. Palmiter, University of Washington; Rousso et al., 2016). Both lines were crossed *to Rosa26YFP* mice (The Jackson Laboratory strain R26R-EYFP, stock 006148). Mice were genotyped by Transnetyx Inc. All experimental animals were housed with littermates of their respective sex before experimental use.

### Electrophysiology

Sexually naive, adult mice (P56–P90) were anaesthetized with isoflurane and killed. Brains were removed and immediately immersed in an ice-cold oxygenated (95% O_2_ and 5% CO_2_) sectioning solution (75 mm sucrose, 10 mm D-glucose, 25 mm NaHCO_3_, 87 mm NaCl, 2.5 mm KCl, 1.0 mm NaH_2_PO_4_, 1.0 mm MgCl_2_ hexahydrate, and 0.5 mm CaCl_2_ dihydrate); 300-μm coronal slices were sectioned on a vibratome (Leica VTS1200) at the level of posterior MeA (bregma −1.56 to −1.94 mm; Franklin and Paxinos, 1997). Slices were collected and placed in oxygen-equilibrated artificial CSF (ACSF) composed of the following: 125.0 mm NaCl, 3.5 mm KCl, 1.0 mm MgCl_2_ hexahydrate, 1.25 mm NaH_2_PO_4_, 2.0 mm CaCl_2_ dihydrate, 26.0 mm NaHCO_3_, and 10.0 mm D-glucose; ∼295–300 mOsm. *Dbx1^cre^*;*RYFP*-positive or *Foxp2^cre^*;*RYFP*-positive neurons were visualized using a epifluorescent microscope (Nikon FN1) with a 450- to 490-nm filter. Whole-cell patch-clamp recordings from YFP-positive fluorescent cells were performed at room temperature with continuous perfusion of ACSF. Signals were acquired on a patch-clamp amplifier (Multiclamp 200B) and digitized at 250 kHz with an A/D converter (DigiDATA1550B). Recordings were performed with glass electrodes pulled on a Sutter P-2000 pipette puller (Sutter Instruments), with ∼3.5-MΩ resistance and filled with a potassium gluconate-based intracellular solution containing the following: 119.0 mm K^+^-gluconate, 2.0 mm Na^+^-gluconate, 6.0 mm NaCl, 2.0 mm MgCl_2_ hexahydrate, 10.0 mm HEPES, 0.9 mm EGTA, 4.0 mm Mg-ATP, 14.0 mm Tris-creatine PO_4_, and 0.3 mm Tris-GTP; pH ∼7.3, ∼285–295 mOsm. Whole-cell patch clamp recordings had an access resistance <30 MΩ at the beginning and end of the experiment or else they were discarded. All measurements of intrinsic and biophysical electrical properties were acquired and analyzed off-line using Clampfit Software 10.6 (Molecular Devices) and GraphPad Prism (GraphPad Software).

### *In situ* hybridization

Animals were intracardially perfused with 4% paraformaldehyde. Brains from perfused animals were collected and suspended in 30% sucrose-PBS solution for ∼24 h. After suspension in sucrose solution, brains were embedded using O.C.T Compound (Fisher HealthCare catalog #23-730-571) and stored at −80°C until cryostat sectioning. Sections at the level of the posterior MeA (bregma −1.56 to −1.94 mm; Franklin and Paxinos, 1997) from cryo-preserved brains were cut at 20 μm with a cryostat (ThermoScientific HM525) and mounted on microscope slides (Fisherbrand catalog #12-550-15).

*In situ* hybridizations were conducted using the RNAscope Multiplex Fluorescent v2. kit following the protocol provided by ACDBio (https://acdbio.com/rnascope%C2%AE-fluorescent-multiplex-assay). This kit permits simultaneous visualization of up to three probes in three separate channels per tissue sample. The protocol was optimized and target retrieval and protease digestion was not performed to preserve tissue quality. Further optimization was achieved by reducing the hydrogen peroxide incubation period from 10 to 5 min. All slides were probed for *Eyfp* (EYFP-C2, catalog #312131-C2) in channel 2 to mark either *Dbx1^cre^;RYFP*-or *Foxp2^cre^;RYFP*-positive cells. The other probes used were: *Hcn2* (Mm-Hcn1, catalog #423651), *Kcna2* (Mm-Kcna-C3, catalog #462811-C3), *Cacna1i* (Mm-Cacna1i, catalog #459781), *Kcnc4* (Mm-Kcnc4-C3, catalog #528091-C3), and *Kcnd2* (Mm-Kcnd2, catalog #452581). Secondary probes used were: CY3 (PerkinElmer TSA Cyanine three Plus Evaluation kit, NEL744E001KT), Alexa Fluor 488 (PerkinElmer TSA Fluorescein Plus Evaluation kit, NEL741E001KT), and CY5 (PerkinElmer TSA Cyanine 5 Plus Evaluation kit, NEL745E001KT). Once the assay was complete, slides were cover-slipped using a DAPI-fluoromount and allowed to dry overnight, then imaged on an Olympus FV1000 confocal microscope at 40× using Olympus Fluoview software version 4.2.1.20. Identification of DAPI+ cells and *Gfp*^+^ and/or ion channel+ positive cells was performed manually with the spots feature in Imaris with each criterion having their own corresponding spot label as follows: (1) cells marked with DAPI had a diameter of 10 μm (±2 μm) and were manually marked centrally with a blue spot. Size was determined using the slice feature in Imaris; (2) cells that contained at least one cluster of *Gfp^+^* signal were manually marked centrally using a green spot; (3) cells that contained at least one cluster of the tested ion channel signal were manually marked centrally using a red spot. Co-localization was performed using the Imaris built-in co-localization feature with an overlap radius of 5 μm. The number of each co-localization was determined using Imaris built-in Statistics tab, which shows the number of co-localizations under its menu.

### Immunohistochemistry

Animals were intracardially perfused with 4% paraformaldehyde. Brains from perfused animals were collected and suspended in 30% sucrose-PBS solution for ∼24 h. After suspension in sucrose solution, brains were embedded in O.C.T Compound (Fisher HealthCare catalog #23-730-571) and stored at −80°C until cryostat sectioning. We used a cryostat to cut sections of posterior MeA (20  μM thick) from cryo-preserved brains (bregma 1.56 to 1.94 mm; Franklin and Paxinos, 1997). Sections were mount on microscope slides.

Sections were washed in TBS to remove excess O.C.T. compound and permeabilized with TBS containing 0.1% Triton X-100 (TBS-T) for 1 h. TBS-T was removed and tissue was then exposed to the primary antibodies (diluted in TBS-T and 4% BSA) for 2 h at room temperature. Primary antibodies used were: mouse anti-Kcnq1/Kv7.1(1:500, catalog #75-081), mouse anti-Kir6.1 (1:200, catalog #75-394), mouse anti-Kir2.1 (1:200, catalog #75-210), mouse anti-Kv1.1 (1:150, catalog #73-007), mouse anti-Kcnt1/Slo2.2/Slack (1:200, catalog #73-051), all from Antibodies Incorporated, and rat anti-GFP (1:100, Nalacai Tesque Inc catalog #04404-84). After incubation with primary antibodies, tissue was rinsed 5× in TBS-T and incubated with secondary antibodies diluted in TBS-T and 4% donkey serum (Jackson ImmunoResearch) for 1 h at room temperature. Secondary antibodies used were: Cy3 donkey anti-mouse (1:500, catalog #715-165-150) and FITC donkey anti-rat (1:1000, catalog #712–095-153) from Jackson ImmunoResearch. Tissue was washed with TBS 5×. Slides were mounted with DAPI-fluoromount and imaged with Zeiss Apotome 2.0 40× objective and 2-μm Z-interval. Images were 3D stacked and analyzed using Imaris Imaging Software, cell counts and colocalizations were done using the “spots” function as described above.

### Data analysis

**Figure 1.**
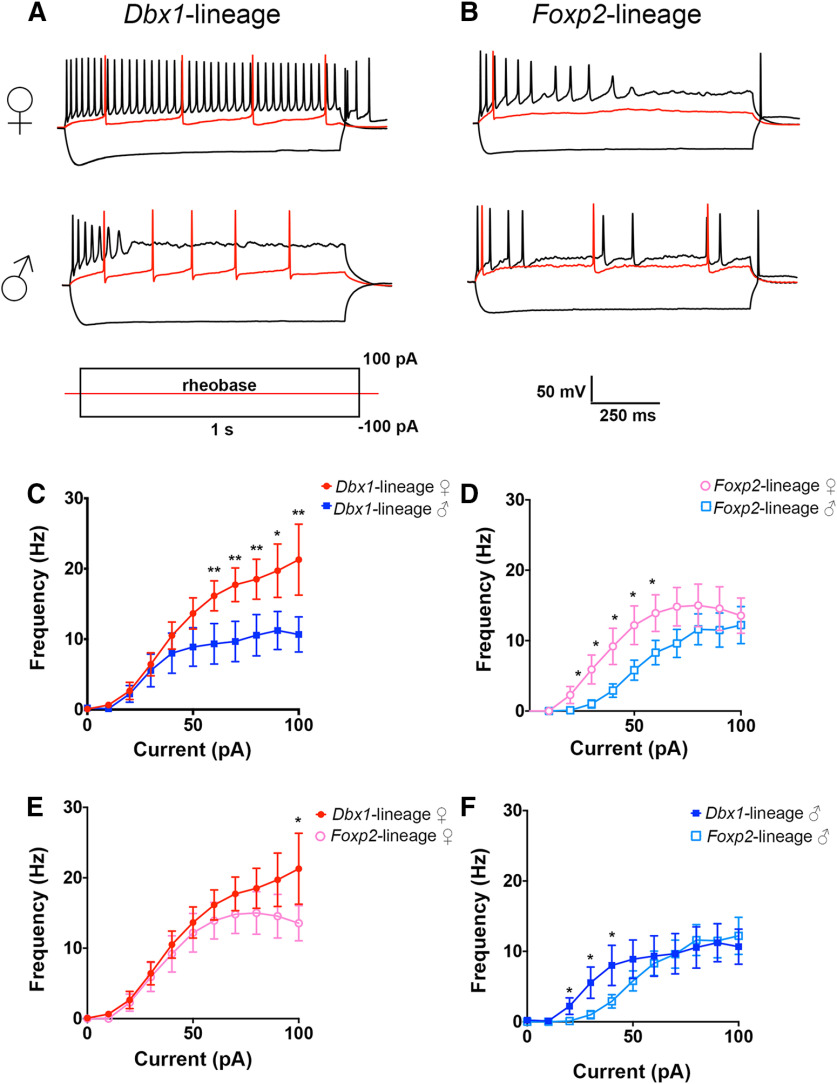
Spiking patterns of MeA *Dbx1-*lineage and *Foxp2*-lineage neurons differ across sex and lineage. Representative spiking patterns of *Dbx1*-lineage (***A***) and *Foxp2*-lineage (***B***) neurons in female and male mice. Traces show voltage responses to a 1-s injection of current at rheobase (red), –100 pA, and +100 pA. Line charts of evoked spike frequency versus current amplitude (***C–F***); *p < 0.05, **p < 0.001.

**Figure 2.**
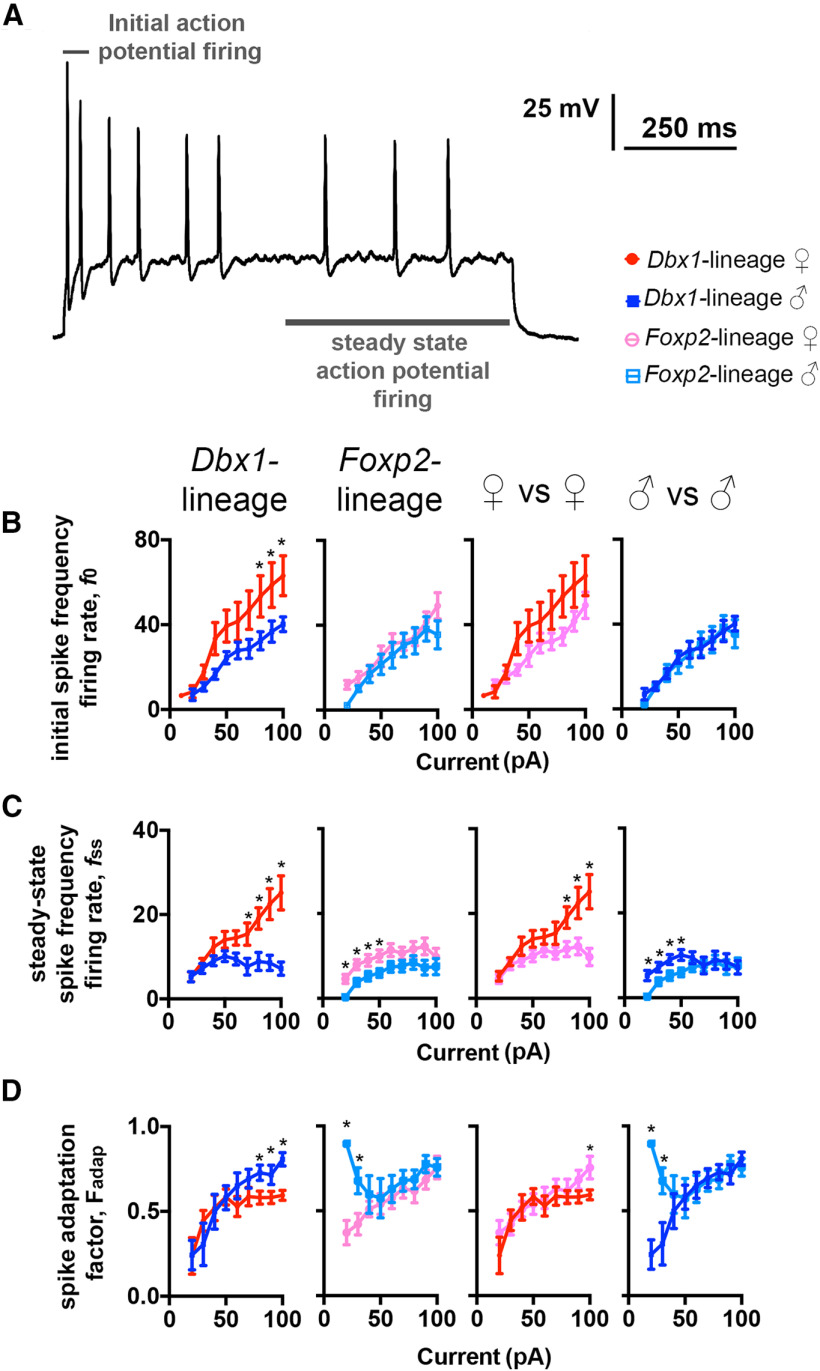
Sex and lineage differences in initial spike frequency, steady-state spike frequency, and spike adaptation. Representative spike trace evoked from a MeA *Foxp2*-lineage neuron from a female mouse demonstrating how initial spike-frequency firing rate (first two spikes) and steady-state spike-frequency firing rate (mean spike rate of last 500 ms) were determined (***A***). Plots of initial spike-frequency firing rate (*f*_0_) versus amplitude of injected current (***B***). Plots of steady-state spike-frequency firing rate (*f*_ss_) versus amplitude of injected current (***C***). Plot of total spike adaptation factor (*F_adap_*) of the spiking frequency versus amplitude of injected current (***D***). *p < 0.05.

**Figure 3.**
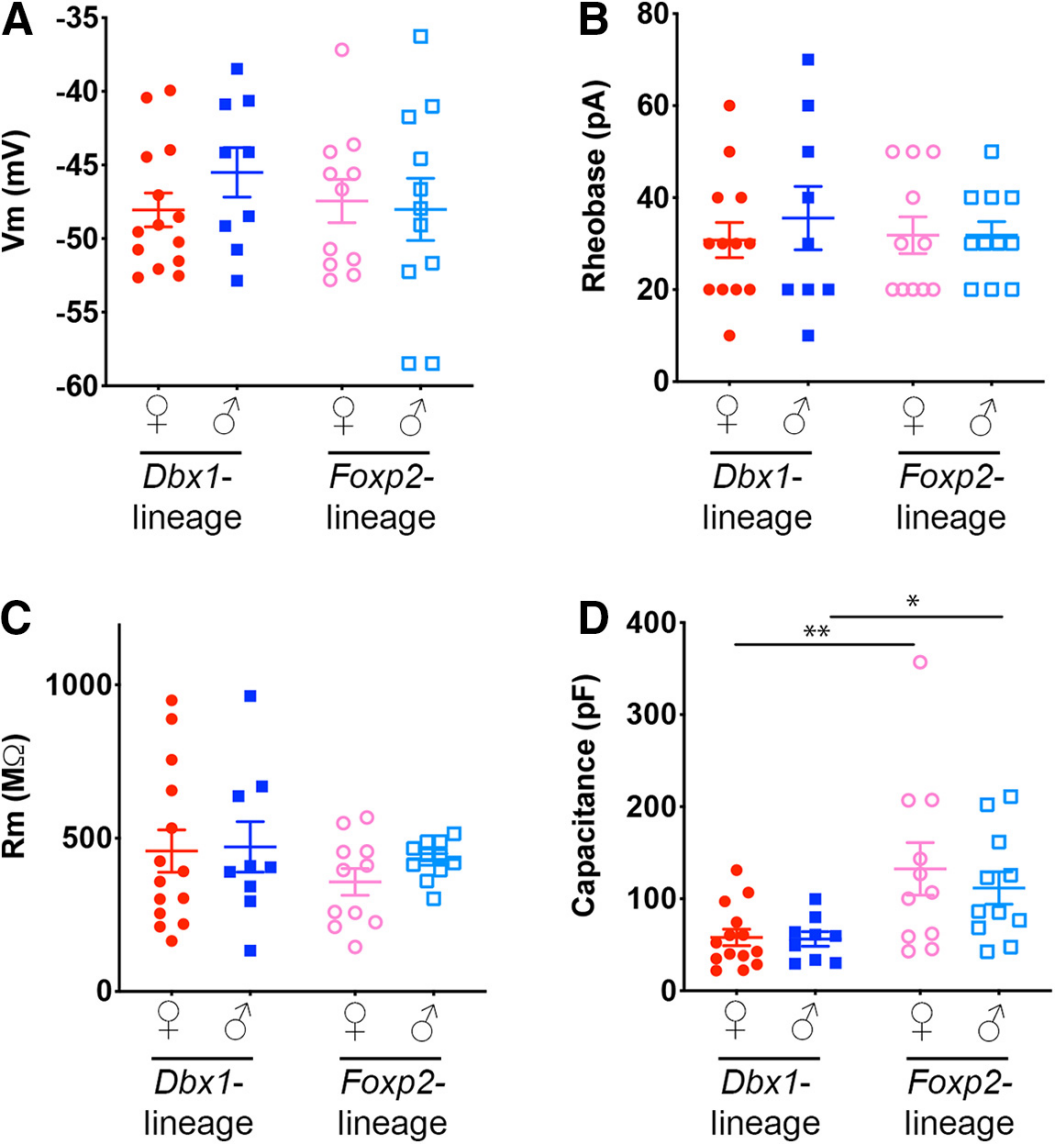
Intrinsic biophysical profiles of MeA *Dbx1-*lineage and *Foxp2-*lineage neurons. Comparison of intrinsic biophysical properties of membrane potential (***A***), rheobase (***B***), membrane resistance (***C***), and capacitance (***D***) across male and female *Dbx1-*lineage and *Foxp2-*lineage neurons. Differences in capacitance were statistically significant; *p < 0.05, **p < 0.001 (see also Extended Data Fig. 3-2). Membrane input resistance and frequency versus voltage described in Extended Data Figure 3-1.

All statistical analyses were performed using GraphPad Prism software. Five different animals were used per experimental group for all electrophysiological experiments (five *Dbx1*^cre^;*RYFP*^+^ females, five *Dbx1*^cre^;*RYFP*^+^ males, five *Foxp2*^cre^;*RYFP*^+^ females, and five *Foxp2^cre^;RYFP^+^* males), and three different animals per group for both the *in situ* hybridization and immunohistochemistry experiments (three *Dbx1*^cre^;*RYFP*^+^ females, three *Dbx1*^cre^;*RYFP*^+^ males, three *Foxp2*^cre^;*RYFP*^+^ females, and three *Foxp2^cre^;RYFP^+^* males). Two-way ANOVA was used for analysis in Figures 1*C–F*, 2*B–E*, 5*D–K* (for both *x*- and *y*-axes values) and Extended Data Figures 6-1*A–E*, 6-3*A–E*, 7-1*A–C*. Following the two-way ANOVA in Figures 1*C–F*, 2*B–D*, Holm–Sidak correction for multiple comparisons was used when comparing means of three or more columns within each row, to correct for type 1 error. One-way ANOVA was used in Figure 3*A–D* and Extended Data Figure 3-1*B*. Following one-way ANOVA in Figure 3*A–D*, and two-way ANOVA in Figures 5*D–K* (for both *x*- and *y*-axes values), Extended Data Figures 6-1*A–E*, 6-3*A–E*, 7-1*A–C*, a multiple comparisons correction was done using Tukey’s test.

10.1523/ENEURO.0035-20.2020.f3-1 Extended Data Figure 3-1 Membrane input resistance and frequency response to voltage. Example of the membrane response (R_in_) to hyperpolarizations (***A***). Arrow indicates the point where we measured the membrane potential used to calculate membrane input resistance. One-way ANOVA shows no difference in calculated input resistance across sex or lineage (***B***). Spiking frequency as a function of voltage calculated from current depolarizations and input resistance (***C***). Download Figure 3-1, TIF file.

10.1523/ENEURO.0035-20.2020.f3-2 Extended Data Figure 3-2 Statistics of intrinsic biophysical properties of *Dbx1-*lineage and *Foxp2*-lineage neurons. Number of neurons recorded, mean, SD, and SEM of the intrinsic values: membrane potential (mV), membrane resistance (MΩ), rheobase and capacitance (pF) of *Dbx1-*lineage and *Foxp2*-lineage MeA neurons in males and females. Download Figure 3-2, DOCX file.

10.1523/ENEURO.0035-20.2020.f6-1 Extended Data Figure 6-1 Immunohistochemistry of voltage-gated ion channel expression in *Dbx1-*lineage and *Foxp2-*lineage neurons in males and females. Immunohistochemistry of GFP+ *Dbx1-*lineage and *Foxp2-*lineage neurons with the voltage gated-ion channels Slo2.2 (females and males pooled together; ***A***), KChip4.1 (***B***), Cav1.2 (***C***), Kv7.1 (***D***), and Kv1.1 (***E***). Two-way ANOVA shows no statistical difference across lineage or sex within lineage. Download Figure 6-1, TIF file.

**Figure 4.**
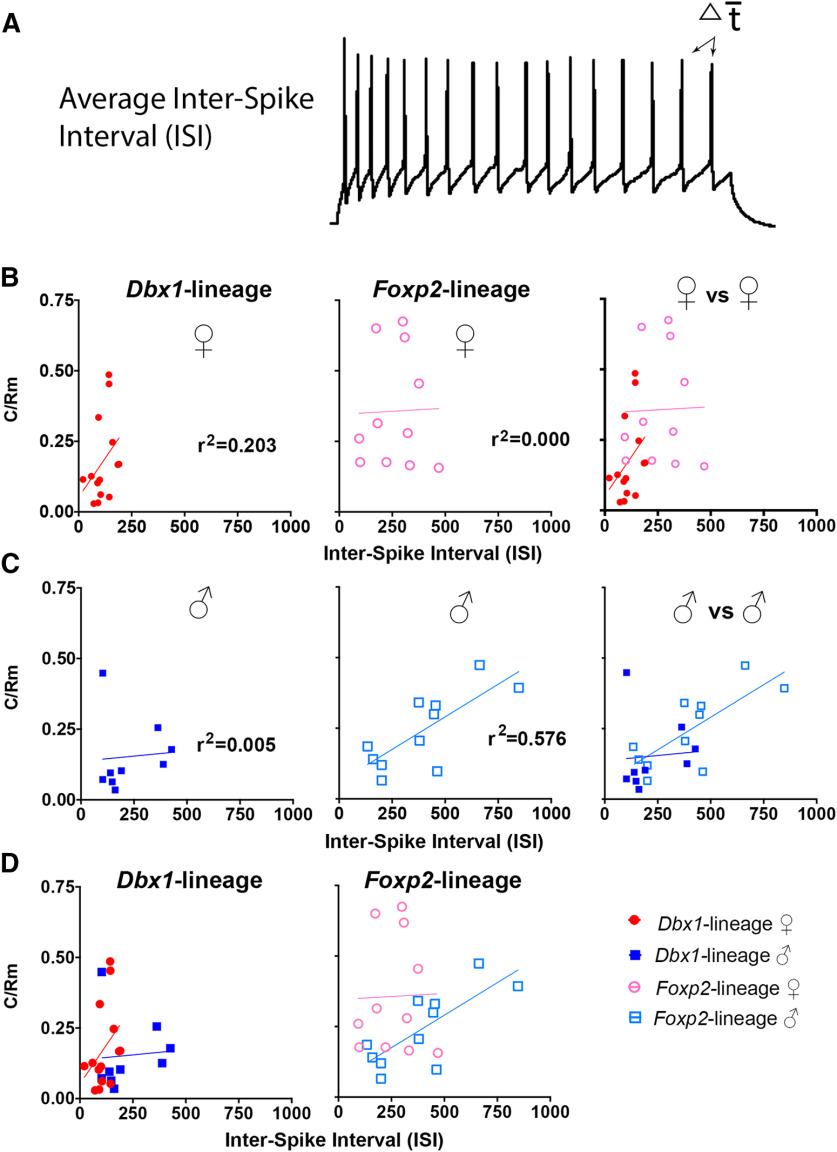
Analysis of stimulation-dependent intrinsic properties of male and female *Dbx1-*lineage and *Foxp2-*lineage neurons. Representative trace of a *Dbx1-*lineage MeA neuron from a female mouse demonstrating how average ISI was measured from all APs evoked during 1-s current injection (***A***). Plots of the ratio of capacitance (C_m_) over membrane resistance (R_m_), C/R_m_, versus ISI (***B–D***).

Instantaneous, or initial, firing frequency (*f_0_*), steady-state firing frequency (*f_ss_*), and adaptation factor (*F_adap_*) were calculated as described previously (Gabbiani and Krapp, 2006). Input resistance of the membrane (R_in_) was calculated as described previously (Ceballos et al., 2016) on steps of 10 pA from 0 to –100 pA. Voltage as a function of current (V_(I)_) was calculated from V = IR, using R_in_ and current steps of 10 pA from 0 to –100 pA. To test the dynamics of the relationship between interspike interval (ISI) and the intrinsic properties of capacitance and membrane resistance (Fig. 4*B–D*), or the stimulation-dependence of the intrinsic values, we used a model of determination coefficient (*R*^2^ model).

**Figure 5.**
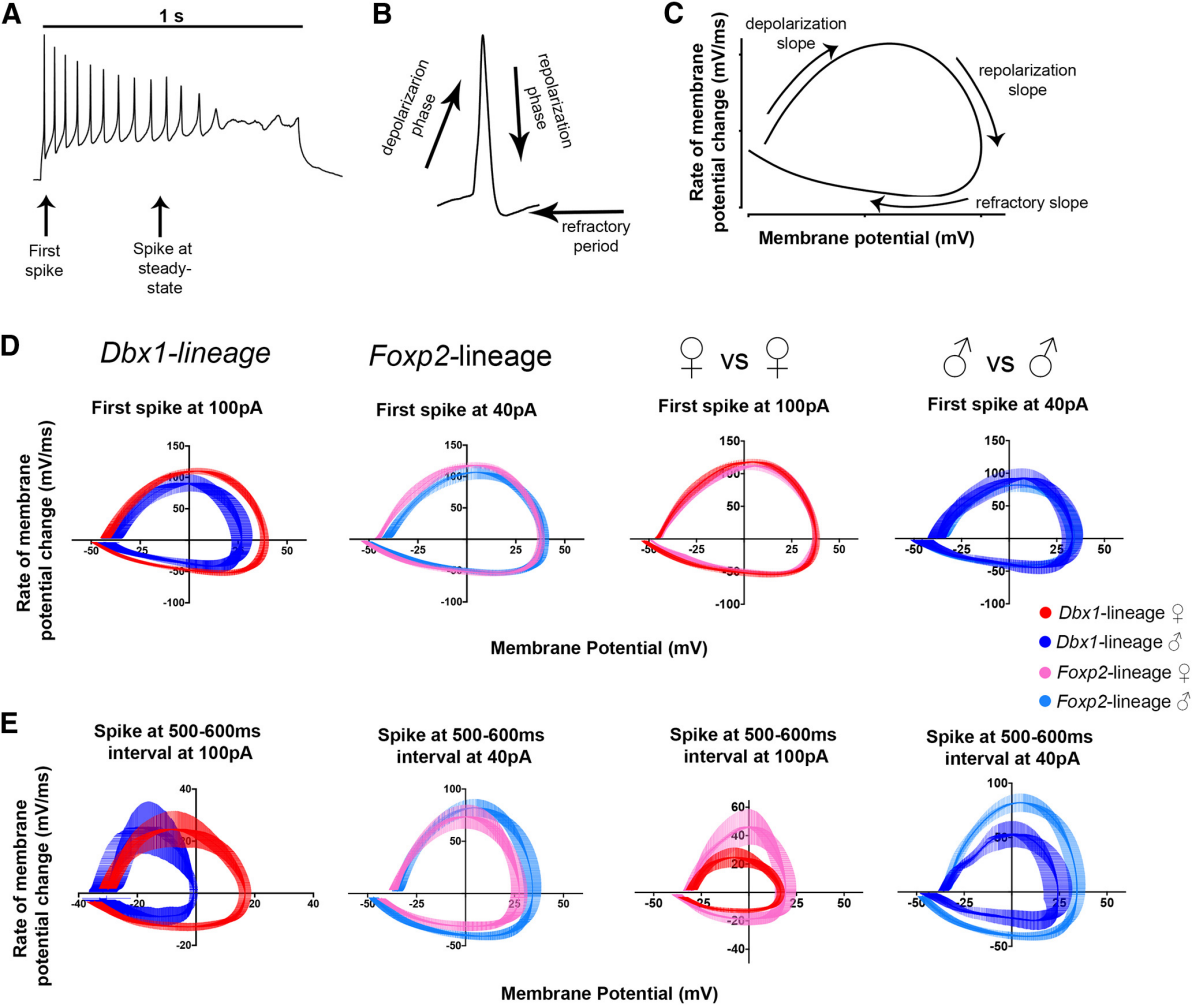
Phase plot analyses of the AP waveform. Representative spike trace from a *Dbx1-*lineage neuron from a male mouse evoked by a 1-s current injection. First spike and steady-state spikes are indicated by arrows (***A***). Schematic of the different phases of typical AP (***B***). Representative phase-plot showing the rate of change of the AP during all three phases of the spike: depolarization, repolarization, and refractory slope (***C***). Phase plots from first spikes of *Dbx1-*lineage and *Foxp2*-lineage neurons at current injections where adaptation differences were identified (***D***). Phase plots of the first spikes evoked at the steady-state during current injection where adaptation differences were identified (***E***).

**Figure 6.**
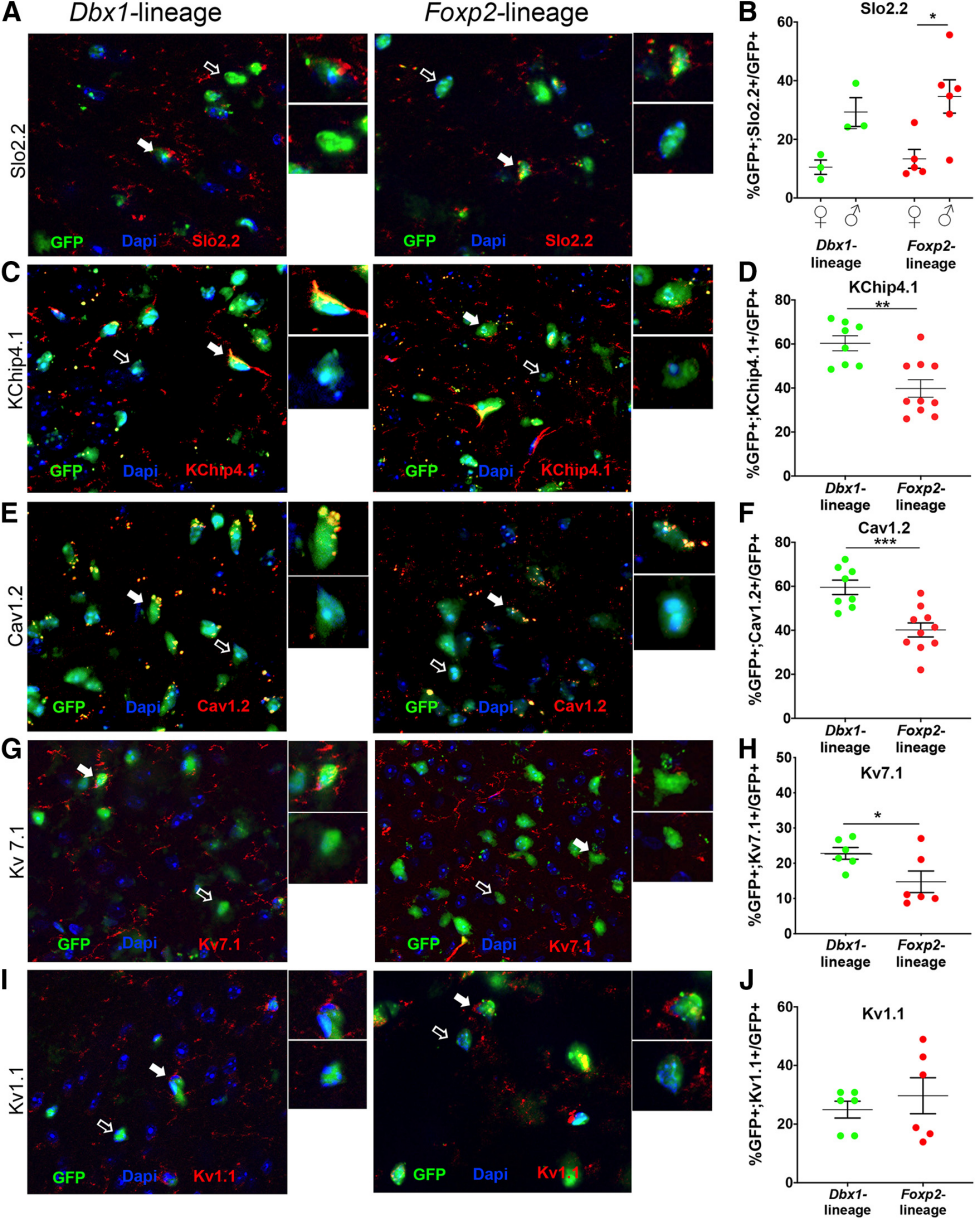
Sex and lineage differences in expression of a subset of voltage-gated ion channels. Immunofluorescent images showing expression of Slo2.2 (red) in GFP+ *Dbx1-*lineage or *Foxp2*-lineage MeA neurons (green; ***A***). Insets show high-power magnification of GFP+ cells colocalized (white arrow) or not colocalized (open arrow) with Slo2.2. Graph of percentages of GFP+ *Dbx1-*lineage and *Foxp2*-lineage neurons in females and males expressing Slo2.2, with significant differences across sex observed in the *Foxp2*-lineage but not the *Dbx1*-lineage (***B***). Immunofluorescent images showing expression of KChip4.1 (***C***), Cav1.2 (***E***), Kv7.1 (***G***), and Kv1.1 (***I***; red) in GFP+ *Dbx1-*lineage or *Foxp2*-lineage MeA neurons (green). Insets show high-power magnification of GFP+ cells colocalized (white arrow) or not colocalized (open arrow) with each channel. Bar graph of percentages of GFP+ *Dbx1-*lineage and *Foxp2*-lineage neurons (male and female grouped) expressing KChip4.1 (***D***), Cav1.2 (***F***), Kv7.1 (***H***), or Kv1.1 (***J***), with significant differences observed across lineage but not sex (see also Extended Data Fig. 6-1) for Kv7.1 and Kir2.1; *p < 0.05, **p < 0.001, ***p < 0.0001. Lineage mRNA expression of additional voltage-gated ion channels is included in Extended Data Figure 6-2, with female and male comparisons in Extended Data Figure 6-3.

**Figure 7.**
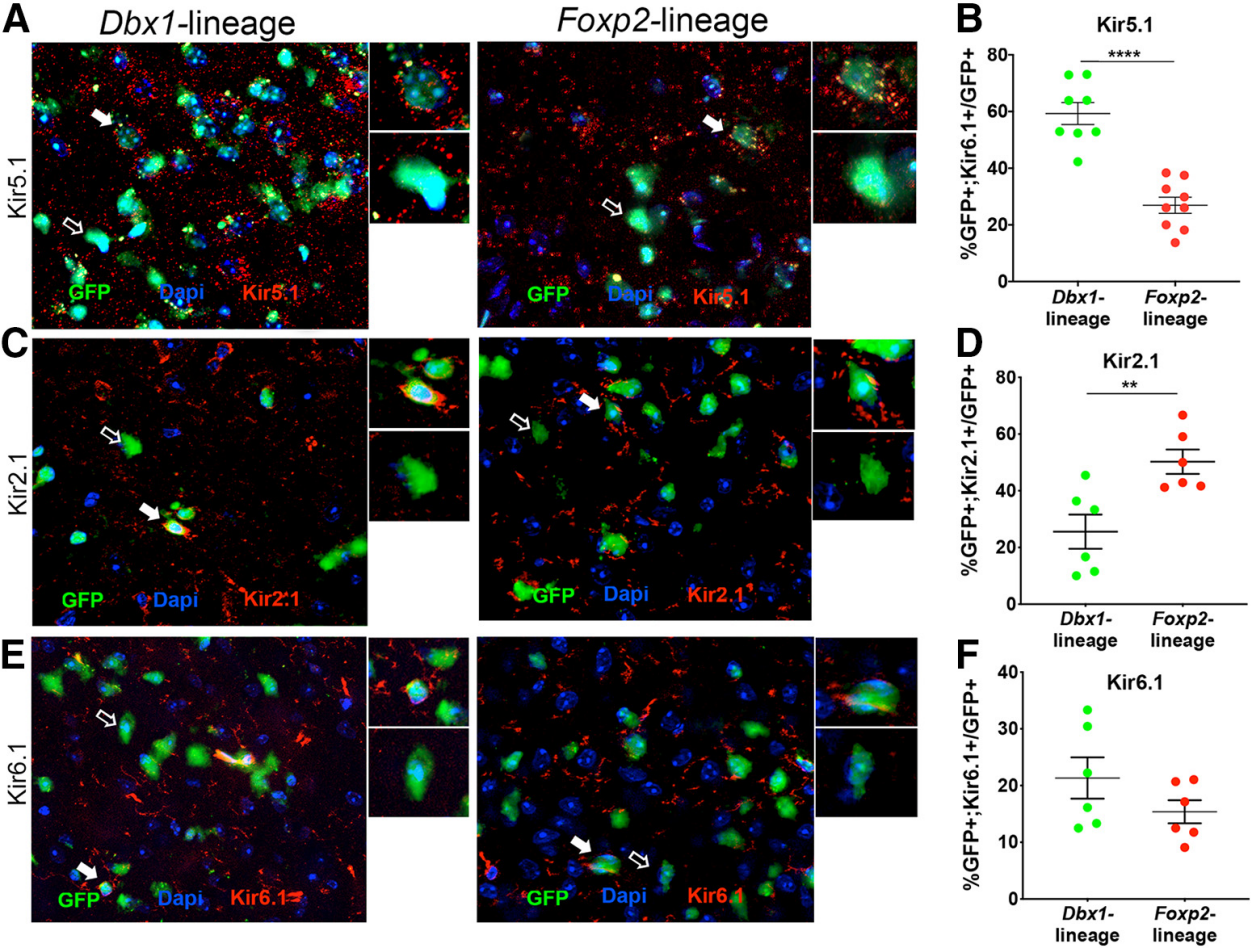
Lineage differences in expression of a subset of inward rectifying K^+^ voltage-gated ion channels. Immunofluorescent images showing expression of Kir5.1 (***A***), Kir2.1 (***C***), and Kir6.1 (***E***; red) in GFP+ *Dbx1-*lineage or *Foxp2*-lineage MeA neurons (green). Insets show high-power magnification of GFP+ cells colocalized (white arrow) or not colocalized (open arrow) with each channel. Bar graph of percentages of GFP+ *Dbx1-*lineage and *Foxp2*-lineage neurons (male and female grouped) expressing Kir5.1 (***B***), Kir2.1 (***D***), or Kir 6.1 (***F***), with significant differences observed across lineage but not sex (see also Extended Data Fig. 7-1) for Kv7.1 and Kir2.1; **p < 0.001.

After testing for sex-specific differences, as described above for Extended Data Figures 6-1*A–E*, 6-3*A–E*, 7-1*A–C*, we pooled the lineage-specific data in Figures 6, 7 and Extended Data Figure 6-2 and analyzed using a *t* test.

10.1523/ENEURO.0035-20.2020.f6-2 Extended Data Figure 6-2 *In situ* hybridization of voltage-gated ion channel genes *in Dbx1-*lineage and *Foxp2-*lineage neurons in males and females. Images of *in situ* hybridization of *Gfp*-expressing *Dbx1-*lineage (left panel) and *Foxp2-*lineage (right panel) neurons co-expressing the voltage-gated ion channel encoding genes *Kcnd2* (***A***), *Cacna1i* (***C***), *Hcn1* (***E***), *Kcna2* (***G***), and *Kcnc4* (***I***). Percentage of pooled male and female *Gfp+* cells expressing *Kcnd2* (***B***), *Cacna1i* (***D***), *Hcn1* (***F***), *Kcna2* (***H***), and *Kcnc4* (***J***). Download Figure 6-2, TIF file.

10.1523/ENEURO.0035-20.2020.f6-3 Extended Data Figure 6-3 Percentages of *Gfp+* cells expressing voltage-gated ion channels. Percentages of co-expression of *Kcnd2* (***A***), *Cacna1i* (***B***), *Hcn1* (***C***), *Kcna2* (***D***), and *Kcnc4* (***E***) from Extended Data Figure 6-2 separated by sex. Two-way ANOVA shows no statistical difference between lineage or sex within lineage in the number of GFP+ cells co-expressing mRNA of either of the measured genes. Download Figure 6-3, TIF file.

10.1523/ENEURO.0035-20.2020.f7-1 Extended Data Figure 7-1 Inward rectifying K^+^ voltage-gated ion channel expression in *Dbx1-*lineage and *Foxp2-*lineages in female and males. Percentages of GFP+ and Kir5.1 (***A***), Kir2.1 (***B***), or Kir6.1 (***C***)-positive cells in *Dbx1-*lineage and *Foxp2-*lineage neurons in females and males; **p < 0.001, ***p < 0.0001. Download Figure 7-1, TIF file.

All recordings were acquired with a 250-kHz sampling rate (Clampex 10.6, and DigiData 1550B, Molecular Devices). Recordings were analyzed using Clampfit 10.6, membrane resistance was measured with a voltage step. As the total number of sample points in a recording is determined by the sampling rate, and the number of points in a digitized AP varied depending on the speed of the speed of the AP, we used the following strategy to make all plots to have the same amount of points facilitate statistical analysis and side-by-side visualization of AP waveforms and subsequent phase plots (Fig. 5*D*,*E*). We generated phase plane plots by independently graphing dV/dT versus V for each AP (Clampfit 10.6) as Cartesian coordinates. Each quadrant was independently plotted starting from the threshold (identified using Clampfit 10.6 threshold detection) as follows:

Cartesian quadrant II = coordinates of the threshold [X_threshold_, Y_threshold_] to [X_1_, Y_max_]

Cartesian quadrant I = (X_1_, Y_max_) to [X_max_, Y = 0],

Cartesian quadrant IV = (X_max_, Y = 0) to [X = 0, Y_min_], and lastly

Cartesian quadrant III = (X = 0, Y_min_) to baseline coordinates ∼[X_min_, Y_1_], where X’s are values in mV, Y’s are values in mV/ms, and [] are inclusive values and () are exclusive values.

*X* and *y*-axes values within each quadrant were resampled to 100 data points by using percentiles. Resampled X and Y values from each quadrant were re-combined and plotted to generate normalized phase plane plots comprised of ∼400 data points. Because each quadrant of the normalized phase-plane plots contained the same number of samples points we were able to generate mean phase-plane plots for APs from group. Descriptive statistics and two-way ANOVAs were calculated using GraphPad Prism.

## Results

The temporal sequence of APs or spiking pattern, is one defining feature of neuronal subclass identity (Petilla Interneuron Nomenclature Group, 2008; Aljadeff et al., 2016; Ha and Cheong, 2017). Spiking patterns are influenced by a number of intrinsic electrophysiological parameters such as resting membrane potential, membrane resistance and cell capacitance (Petilla Interneuron Nomenclature Group, 2008; Boada, 2013; Li and Tsien, 2017; Tapia et al., 2018; Bomkamp et al., 2019). To characterize the spiking patterns of MeA *Dbx1-*lineage and *Foxp2*-lineage neurons in females and males, we performed whole-cell patch clamp on YFP-expressing neurons in *Dbx1^cre^*;*RYFP* and *Foxp2^cre^*;*RYFP* mice. We used an evoked spiking protocol and recorded membrane potential during stepwise current injection from −100 to 100 pA in 10-pA intervals (Fig. 1*A*,*B*).

During high-amplitude current injection, *Dbx1*-lineage neurons in females (*n* = 14) discharged more spikes than in males (*n* = 9; two-way ANOVA *p* < 0.0001, Holm–Sidak correction for multiple comparisons 60 pA *p* = 0.032, 70 pA *p* = 0.011, 80 pA *p* = 0.008, 90 pA *p* = 0.012, 100 pA *p* = 0.002; Fig. 1*C*), whereas *Foxp2*-lineage neurons in females (*n* = 11) discharged more spikes than in males (*n* = 11) during low-amplitude current injection (two-way ANOVA *p* < 0.0001, Holm–Sidak correction for multiple comparisons 30 pA *p* = 0.043, 40 pA *p* = 0.018, 50 pA *p* = 0.026, 60 pA *p* = 0.023, 70 pA *p* = 0.046; Fig. 1*D*). When comparing spiking patterns across lineages, we found that *Dbx1*-lineage neurons discharge more spikes than *Foxp2*-lineage neurons in females, but only at the highest amplitude stimulus (two-way ANOVA *p* < 0.0001, Holm–Sidak correction for multiple comparisons *p* = 0.029; Fig. 1*E*). In contrast to female mice, *Dbx1-*lineage neurons in male mice spiked more than *Foxp2*-lineage neurons in males during low rather than high-amplitude current injection (two-way ANOVA *p* < 0.0001, Holm–Sidak correction for multiple comparisons 30 pA *p* = 0.043, 40 pA *p* = 0.043, 50 pA *p* = 0.048; Fig. 1*F*). Collectively, these data reveal that the spike frequency of *Dbx1-*lineage and *Foxp2*-lineage neurons differs by lineage, sex, and stimulus strength.

The progressive slowing of spike frequency is referred to as spike-frequency adaptation (Tripathy et al., 2014). Spike-frequency adaptation plays an important role in neural coding (Ha and Cheong, 2017). Thus, we evaluated spike-frequency adaptation in *Dbx1-*lineage and *Foxp2*-lineage cells by deriving a spike-frequency adaptation factor (*F_adap_*) obtained from the initial frequency firing-rate (*f_0_*) and the steady-state frequency firing-rate (*f_ss_*; Fig. 2; Gabbiani and Krapp, 2006; see Materials and Methods). We calculated *f_0_* from the first two evoked spikes, and *f_ss_* from the mean spike rate during the steady-state, defined here as the last 500 ms of current injection (Fig. 2*A*). We found that *Dbx1*-lineage neurons in females displayed a higher *f_0_*than males (Fig. 2*B*), while there was no sex difference in the *f_0_* of *Foxp2*-lineage neurons (Fig. 2*B*). We also did not detect differences between the *f_0_* of neurons in females or males across lineages (Fig. 2*B*). During steady state firing, however, we found that *f_ss_* increased in direct proportion to the amplitude of the current injected into *Dbx1*-lineage neurons in females but not males (Fig. 2*C*). In contrast, *Foxp2*-lineage neurons in females exhibited a higher *f*_ss_ than males but only when the amplitude of the injected current was <70 pA. When we compared *f_ss_* across lineages in female mice, we found that *Dbx1*-lineage neurons had a higher *f_ss_* than *Foxp2*-lineage neurons and that the *f_ss_* in *Dbx1*-lineage neurons was more sensitive to increases in current amplitude. *Dbx1*-lineage neurons in males had a higher *f_ss_* than *Foxp2*-lineage neurons in males, but only during low-amplitude current injection. We used the *f_0_* and *f_ss_*values to calculate the adaptation factor (*F_adap_*), which measures dynamic changes in spike frequency as a function of current amplitude (Fig. 2*D*). Across sex, high-amplitude current injection to *Dbx1*-lineage neurons in males exhibited a larger *F_adap_* than *Dbx1*-lineage neurons in females. In contrast, low-amplitude current injection into *Foxp2*-lineage neurons in males exhibited a larger *F_adap_* than *Foxp2*-lineage neurons in females. Across lineages, the *F_adap_* of neurons *Foxp2*-lineage neurons in females was greater than *Dbx1-*lineage in females, but only during the highest amplitude of current injection. *Foxp2*-lineage neurons in males also displayed a higher *F_adap_* than *Dbx1*-lineage neurons in males but only during low-amplitude current injections. Collectively, these data demonstrate that spike-frequency adaptation correlated with neuronal lineage and sex with females having a lower adaptation factor than males and *Foxp2*-lineage neurons displaying a higher adaptation factor than *Dbx1*-lineage neurons. More broadly, our data reveal that there are sex and lineage differences in multiple dynamic parameters of the AP. Interestingly, these differences manifest at different levels of current stimulation with *Dbx1*-lineage neurons displaying sex differences only at higher levels of current stimulation than *Foxp2*-lineage neurons.

The intrinsic biophysical properties of a neuron, such as membrane potential, rheobase, membrane resistance, and membrane capacitance contribute to firing properties as well as how neurons function within networks (Brown et al., 2019). Therefore, we next defined the intrinsic biophysical profiles of *Dbx1-*lineage and *Foxp2*-lineages in both females and males (Fig. 3; Extended Data Figs. 3-1, 3-2). In contrast to our above experiments (Figs. 1, 2), which revealed sex and lineage differences in spiking pattern and adaptation, we found no changes in the static properties of membrane potential, rheobase or membrane resistance (Fig. 3*A–C*; Extended Data Fig. 3-1). However, we found that *Foxp2-*lineage neurons (in both males and females) had higher capacitance than *Dbx1*-lineage neurons (Fig. 3*E*). This suggests that that *Foxp2*-lineage neurons may have greater cell surface than *Dbx1*-lineage neurons.

As there were no differences in static intrinsic properties, aside from capacitance, we next explored whether stimulation-dependent changes to intrinsic properties could explain the difference in spiking patterns shown in Figures 1, 2. To determine whether dynamic changes to intrinsic properties differed across sex and/or lineage, we plotted the ratio of capacitance (C_m_)/membrane resistance (R_m_) versus the average ISI from each trace (from Fig; 1; Extended Data Fig. 3-2). Our rationale was that if the ratio of C_m_/R_m_ correlated with the ISI, it would demonstrate that the intrinsic properties of the neuron are dynamic and dependent on stimulation. This would suggest that the difference in spiking patterns are due to voltage-dependent changes to intrinsic properties. However, if the ratio of C_m_/R_m_ did not correlate with the ISI, this would suggest that intrinsic properties are static and voltage independent. The capacitance (C_m_) and membrane resistance (R_m_) showed a weak but positive correlation to ISI in *Dbx1-*lineage neurons from females (*r*^2^ = 0.203; Fig. 4*B*). This is in contrast to *Foxp2*-lineage neurons in females which did not show a correlation to ISI (*r*^2^ = 0.000; Fig. 4*C*). In contrast to *Dbx1*-lineage neurons in females, there was no correlation in the ratio of C_m_/R_m_ to ISI in *Dbx1-*lineage neurons in males (*r*^2^ = 0.0003; Fig. 4*B*). *Foxp2*-lineage neurons in males showed a stronger correlation between the ratio of C_m_/R_m_ to ISI (F, *r*^2^ = 0.576; Fig. 4*C*). We also observed sex differences within each lineage (Fig. 4*D*). Together, this analysis demonstrates that there are both sex and lineage differences in the stimulation-dependent changes in intrinsic properties. This may provide a biophysical mechanism to explain the dramatic differences in spiking shown in Figures 1, 2.

The membrane capacitance (C_m_) and the membrane resistance (R_m_) determine how fast the cell membrane potential responds to ion flux (Golowash and Nadim, 2014). We observed sex and lineage differences in these ratios upon stimulation (Fig. 4), indicating a mechanistic role for differences in ion channel expression, regulation, or composition in modulating the observed spiking differences. Different families of ion channels generate distinct aspects of the AP waveform. As the waveform of spikes evoked from *Foxp2*-lineage and *Dbx1*-lineage neurons was influenced by stimulus intensity (Fig. 1), we next analyzed the waveforms of the initial spike and the first spike during steady-state firing at the current injection amplitudes that generated the largest difference in spike-frequency adaptation (Fig. 5*A*; as described in Fig. 2). We used a phase-plot model (Fig. 5*B*), which allows quantitative examination of distinct phases of the AP and provides insights into potential ion channels that modulate those specific phases (FitzHugh, 1961; Drion et al., 2012; Fig. 5*C*). In *Dbx1*-lineage neurons, we observed sex differences in the repolarization and refractory slopes of the initial evoked AP (Fig. 5*D*; see Materials and Methods for all statistics). We also observed sex differences in the repolarization slope of *Dbx1-*lineage neurons, which were greater during steady-state spiking than during the initial AP (Fig. 5*E*). In *Foxp2*-lineage neurons, we observed sex differences during a portion of the repolarization slope and refractory slope, but only during steady state firing (Fig. 5*D*,*E*). We also observed lineage differences in the waveforms of the first spikes evoked during steady-state firing, but not during initial firing (Fig. 5*D*). In females, lineage differences were observed during the depolarization and repolarization slopes. In males, lineage differences were observed during all phases of the AP waveform. Thus, modulation of AP components in *Dbx1-*lineage and *Foxp2*-lineage neurons differ across both sex and lineage.

**Figure 8.**
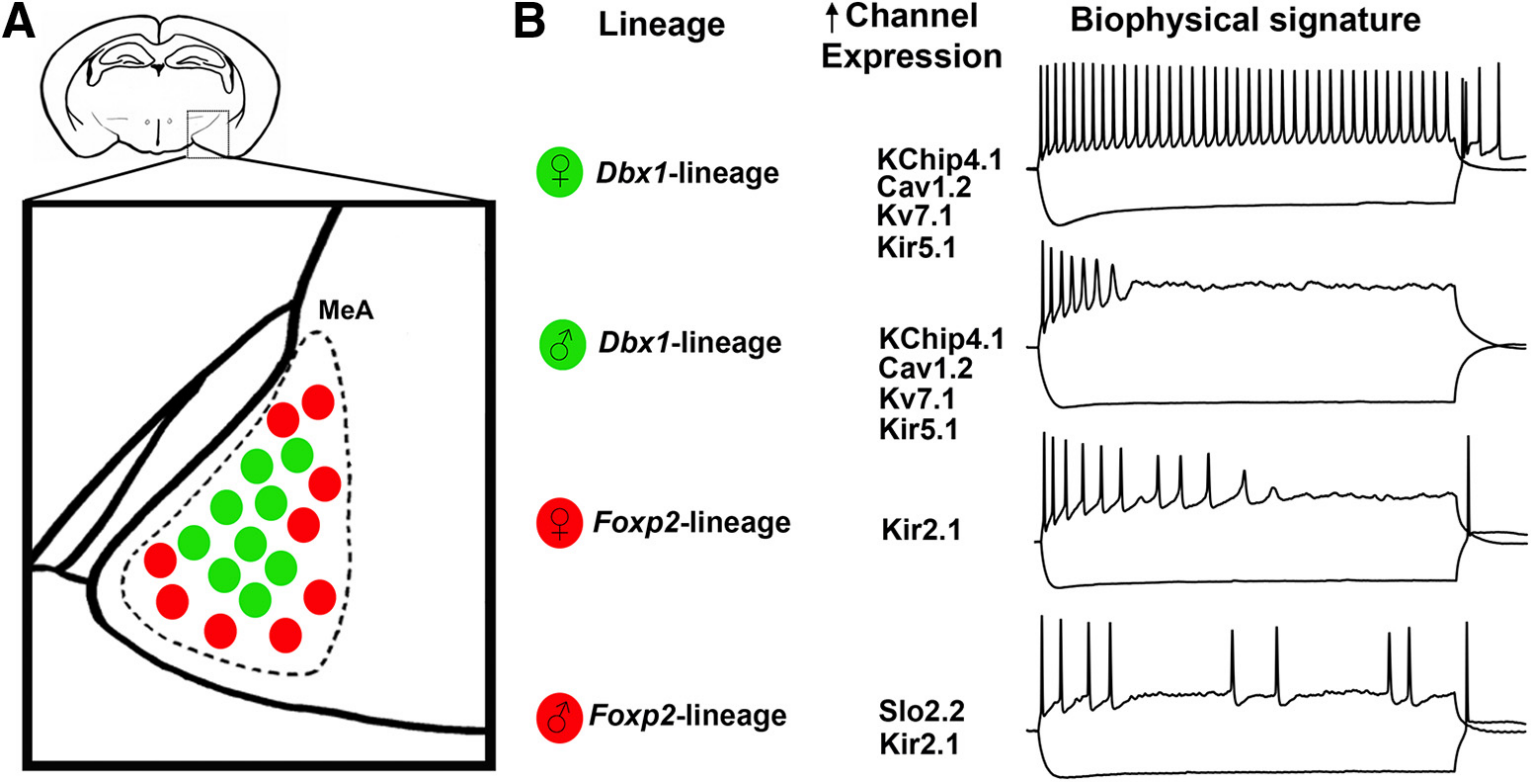
Summary of findings. Schematic of *Dbx1-*lineage (green) and *Foxp2*-lineage (red) neurons in the adult mouse MeA (***A***), voltage-gated ion channels with highest expression in each respective population (lineage or sex), and their corresponding sex-specific and lineage-specific biophysical signatures (***B***).

The most consistent differences in the AP waveforms across sex and lineage occurred during the repolarization phase (Fig. 5). Repolarization of the neuronal membrane and spike adaptation are largely modulated by voltage gated potassium (K^+^) and calcium (Ca^2+^) channels (Hille, 1991; Miller, 1992; Pathak et al., 2016; Ha and Cheong, 2017). We first referenced the *Allen Brain Atlas: Adult Mouse Brain* (Allen Mouse Brain Atlas 2019; Lein et al., 2007) to identify genes expressed in the MeA that encode K^+^ and Ca^2+^ channels. We then explored sex and lineage expression of 13 of these K^+^ and Ca^+^ channel subtypes known to regulate the AP repolarization phase: KChip4.1, Ca_v_1.2, K_v_7.1, K_v_1.1, K_ir_6.1, K_ir_5.1, K_ir_2.1, Slo2.2, *Kcnd2*, *Cacna1i*, *Hcn1*, *Kcna2*, and *Kcnc4* either by immunofluorescence (Figs. 6, 7; Extended Data Figs. 6-1, 7-1) or *in situ* hybridization (Extended Data Figs. 6-2, 6-3) based on reagent availability. Of the proteins and mRNA transcript expression assessed, we found sex differences in expression of Slo2.2 in the *Foxp2*-lineage (ANOVA, *p* = 0.020; Fig. 6*B*), lineage differences in the K^+^ and Ca^2+^ channels; KChip4.1, K_v_7.1, Ca_v_1.2, (*t* test, *p* = 0.0016, *p* = 0.008, and *p* = 0.0007, respectively; Fig. 6*D*,*G*,*I*), and in the K^+^ inward rectifying channels K_ir_ 5.1 and K_ir_2.1 (*t* test, *p* = 0.00001 and *p* = 0.043, respectively; Fig. 7*B*,*D*; Figs. 6, 7; Extended Data Figs. 6-1, 6-2, 6-3, 7-1; see summary in Fig. 8).

## Discussion

Sex differences in cell morphology, dendritic complexity, and cell size have been well characterized in the MeA (Cooke et al., 2007; Dall’Oglio et al., 2008; Unger et al., 2015). In addition, there are sex differences in the expression of a number of molecular markers especially hormone pathway genes (Jasnow et al., 2007; Xu et al., 2012; Chen et al., 2019; Gegenhuber and Tollkuhn, 2019). Moreover, the MeA displays sex-specific responses to olfactory cues as revealed by cFos staining or *in vivo* electrophysiological recordings (Bergan et al., 2014; Yang and Shah, 2014; Carvalho et al., 2015; Ishii et al., 2017; Li and Dulac, 2018). However, how these characteristics translate to sex differences in behavioral responses is unknown. A critical step to bridging this gap is to understand whether and how biophysical profiles, especially AP firing, differ across sex. In this study, we characterized spiking patterns, membrane properties, spike adaptation, and dynamic AP changes of two major classes of output neurons in the female and male MeA previously defined by us by their developmental expression of the transcription factors, Dbx1 or Foxp2 (Hirata et al., 2009; Carney et al., 2010; Lischinsky et al., 2017). Here, our multidimensional analyses reveal sex-specific and lineage-specific electrophysiological and ion channel expression profiles of these neuronal populations. Thus, our findings provide a physiological characterization of two neuronal populations that, at the individual neuron level, potentially contribute to how the male and female MeA may process social and non-social cues that trigger innate responses.

Previous data from our laboratory revealed that major populations of MeA inhibitory output neurons are derived from progenitor cells in the preoptic area (POA) of the embryonic ventral telencephalon. The transcription factors Dbx1 and Foxp2 delineate two of these progenitor populations (Hirata et al., 2009; Carney et al., 2010; Lischinsky et al., 2017). This embryonic parcellation by transcription factor expression persists into adulthood where *Dbx1-*lineage and *Foxp2*-lineage neurons possess broad electrophysiological and molecular differences (Lischinsky et al., 2017). Previous electrophysiological characterization and molecular profiling studies by us and others have revealed that the MeA is comprised of as many as 19 different neuronal subtypes (Bian, 2013; Keshavarzi et al., 2014; Lischinsky et al., 2017; Chen et al., 2019; Canteras et al., 2019). Recent molecular profiling and electrophysiological studies have explored whether this diversity extends to sex (Chen et al., 2019; Dalpian et al., 2019). These studies revealed sex differences in both gene expression (Chen et al., 2019) and broad aspects of intrinsic electrophysiological profiles (Dalpian et al., 2019). However, the neuronal subtypes in which these differences were observed were not explored nor was exploration into potential mechanism underlying biophysical differences. Here, our developmental-based molecular markers provided us a means to study sex differences in identifiable subtypes and explore putative biophysical mechanisms underlying sex and lineage differences.

One powerful means to explore biophysical mechanisms underlying spiking dynamics is AP phase plot analyses. Our detailed phase plot analyses revealed sex and lineage differences in spike dynamics (Fig. 5), implying contributions of different ion channel subclasses. Our molecular analyses validated the expression of several candidate ion channels within *Dbx1-*lineage and *Foxp2*-lineages. We revealed that a substantial portion of *Dbx1*-lineage neurons express KChip4.1, Ca_v_1.2, K_v_7.1, and K_ir_5.1 at a greater proportion than in the *Foxp2*-lineage. KChip4 is a native of the K_v_4 complex, which controls repolarization of the membrane after AP (Holmqvist et al., 2002; Kim et al., 2005). Ablation of K_v_4 from hippocampal neurons results in higher spiking frequency (Carrasquillo et al., 2012). K_v_7.1 is a voltage-gated, non-inactivating potassium channel that that regulates “phasic firing” (Greene and Hoshi, 2017), spike adaptation, and spiking patterns by attenuating hyperexcitability (Jentsch, 2000; Robbins, 2001; Hu et al., 2007). K_ir_5.1 is a pH-sensitive inward rectifying channel that regulates potassium flux and neuronal spiking (D'Adamo et al., 2011; Puissant et al., 2017). Ca_v_2.2 encodes for the Ca_v_1.2 voltage-gated ion channel which regulates excitability and spiking (Michailidis et al., 2014). Cav1.2 increases the activation of small conductance calcium activated potassium currents (I_sk_) which flattens the frequency-current curve (Mäki-Marttunen et al., 2016). By contrast, a higher proportion of *Foxp2*-lineage neurons express K_ir_2.1, an inward rectifying potassium channel that rapidly repolarizes excitable membranes (Lu, 2004) by allowing a large inward K^+^ flux (Hibino et al., 2010). *Dbx1-*lineage and *Foxp2*-lineage neurons clearly express distinct, and non-overlapping patterns of ion channels. All of these channels have well-characterized roles in modulating AP firing properties, and could thus contribute to the different firing frequencies of *Dbx1-*lineage and *Foxp2*-lineage neurons. However, how these channels specifically regulate the lineage differences in AP firing remains to be explored.

Our phase plot analysis also reveals sex differences in spike waveform during the steady-state, most notably at the repolarization phase, which is also mediated by voltage-gated ion channels. Our molecular analysis reveals *FoxP2-*lineage neurons in male express a higher proportion of the Slo2.2 channel than females. Slo2.2, also known as Slack, is an outward K^+^ rectifying channel that plays a major role in control of neuronal excitability (Joiner et al., 1998). Ablation of this channel in sensory dorsal ganglion neurons results in increased spiking frequency and changes in both depolarization and repolarization phases (Martinez-Espinosa et al., 2015). As male *Foxp2*-lineage neurons exhibit lower spiking frequency and different AP phases than female *Foxp2*-lineage neurons, the sex differences in expression of Slo2.2 provides a suitable molecular candidate for these observed differences. A recent RNA-seq profiling study explored sex differences in the MeA (Chen et al., 2019) and found that males express higher mRNA levels of the K^+^ channel *Kcnip4* and the Ca^2+^ channel *Cacna1c*. *Kcnip4* encodes for KChip4, and *Cacna1c* encodes for Ca_v_1.2, candidates which we tested here. However, we did not detect sex differences in the expression of either channel in *Dbx1-*lineage or *Foxp2*-lineage, suggesting that the previous reported sex differences may correspond to other populations of MeA neurons. It is important to note that *Dbx1-*lineage and *Foxp2*-lineage neurons likely express many additional ion channels that may synergistically regulate membrane properties. The specific mechanism by which combinations of ion channels modulate sex or lineage differences in AP firing remains to be elucidated. Nevertheless, our data establishe a putative relationship between the expression and function of voltage-gated ion channels that can generate distinct spiking patterns in adult MeA neurons.

The male and female MeA respond differently to male and female olfactory cues (Jasnow et al., 2007; Xu et al., 2012; Chen et al., 2019; Gegenhuber and Tollkuhn, 2019). However, how olfactory information in the MeA is encoded at the neuron and circuit level remains poorly understood. AP spiking patterns and spike-frequency adaptation are critical parameters in determination of neuronal coding (Kreiman, 2004; Peron and Gabbiani, 2009a; Li and Tsien, 2017). For example, neurons can convey information by transmitting spikes at a particular frequency, and, thus, differences in spiking may drive differences in neuronal coding in the computational functions of the brain (Aljadeff et al., 2016; Li and Tsien, 2017; Ha and Cheong, 2017). This is an important characteristic because information transfer, through spiking patterns, relates both to the nature of the inputs the neuron receives as well as their readout (Peron and Gabbiani, 2009a). Thus, spiking-frequency adaptation can be determined by two non-mutually exclusive mechanisms: (1) inputs coming to the neuron and/or (2) the neuronal intrinsic properties (Peron and Gabbiani, 2009b). Our results demonstrate that in both lineages, males and females differ not only in their spiking pattern and adaptation properties, but also in how their intrinsic properties can change throughout stimulation. How these novel sex differences in AP properties relate to sex-specific behaviors is unknown. This observation identifies one mechanism for regulating spike frequency in a sex-specific manner but does not exclude sex differences in inputs. Most inputs to the MeA come from the accessory olfactory bulb (Cádiz-Moretti et al., 2016); however, it is unclear whether they are sexually dimorphic in anatomy and/or synaptic organization.

Differences in gene expression in the MeA and behavioral repertoires between males and females appear to be largely driven by hormonal control (Cooke et al., 1999; Yang and Shah, 2014; Li and Dulac, 2018; Krolick et al., 2018; Gegenhuber and Tollkhun, 2020). For example, estrogen signaling in the MeA controls sex stereotypical behaviors in both sexes in rodents and many other vertebrates (for review, see McCarthy, 2008). In addition, there are sex differences in hormonally regulated numbers of MeA aromatase^+^ neurons that mediate male aggression (Wu et al., 2009; Unger et al., 2015). Hormonal regulation has also been shown to modulate voltage-gated channel function, which may relate to the sex differences uncovered by our phase plot and expression analyses. Estrogen regulates voltage-gated ion channel expression in smooth muscle, dorsal ganglion and hypothalamic gonadotropin-releasing hormone (GnRH) neurons (Farkas et al., 2007; Du et al., 2014; Shi et al., 2015; Kow and Pfaff, 2016). In the bird auditory system, estrogens increase neuronal responsiveness by suppressing inhibitory transmission (Tremere et al., 2009), while local estrogen levels rapidly change burst firing of single auditory neurons (Remage-Healey et al., 2010). In addition to estrogens, androgens also affect the biophysical properties of neurons by modulating the expression of both voltage-gated and ligand-gated ion channels (Penatti and Henderson, 2009). For example, chronic exposure to androgenic anabolic steroids increases GABAergic transmission in the mouse hypothalamic pre-optic area (Penatti et al., 2009). Additionally, the estrous cycle may play a role in synaptic related molecular changes as well as intrinsic synaptic properties in females (Hirsch et al., 2018; Dalpian et al., 2019). Thus, hormones are well positioned to play a role in determination or modulation of male and female biophysical differences in the MeA. How and when sex hormones shape MeA neuronal firing properties and how this relates to network function will be an important and interesting area of future investigation.

## Synthesis

Reviewing Editor: Cheryl Sisk, Michigan State University Decisions are customarily a result of the Reviewing Editor and the peer reviewers coming together and discussing their recommendations until a consensus is reached. When revisions are invited, a fact-based synthesis statement explaining their decision and outlining what is needed to prepare a revision will be listed below. Note: If this manuscript was transferred from JNeurosci and a decision was made to accept the manuscript without peer review, a brief statement to this effect will instead be what is listed below.

Authors have adequately addressed the concerns raised in the original review.
