# Supplementary material for: Sex Differences in Biophysical Signatures across Molecularly Defined Medial Amygdala Neuronal Subpopulations
Source: eNeuro. 2020 Jul 2;7(4):ENEURO.0035-20.2020. doi: 10.1523/ENEURO.0035-20.2020 (PMC7333980; doi:10.1523/ENEURO.0035-20.2020)
Supplement: Extended Data Figure 3-2 — Statistics of intrinsic biophysical properties of Dbx1-lineage and Foxp2-lineage neurons. Number of neurons recorded, mean, SD, and SEM of the intrinsic values: membrane potential (mV), membrane resistance (MΩ), rheobase and capacitance (pF) of Dbx1-lineage and Foxp2-lineage MeA neurons in males and females. Download Figure 3-2, DOCX file. [file enu-eN-NWR-0035-20-s02.docx]

| Membrane Potential (Vm) | | | |  |
| --- | --- | --- | --- | --- |
| Descriptive statistics | *Dbx1*-lineage | *Dbx1*-lineage | *Foxp2*-lineage | *Foxp2*-lineage |
| Number of cells | 14 | 9 | 1 1 | 1 1 |
| Mean | -48.06 | -45.5 | -47.45 | -48.02 |
| Std. Deviation | 4.293 | 5.027 | 4.876 | 7.013 |
| Std. Error of Mean | 1 .147 | 1 .676 | 1 .47 | 2.1 14 |
| Membrane Resistance (M **Ω**) | | | |  |
| Descriptive statistics | *Dbx1*-lineage | *Dbx1*-lineage | *Foxp2*-lineage | *Foxp2*-lineage |
| Number of cells | 14 | 9 | 1 1 | 1 1 |
| Mean | 458 | 471 .5 | 357.4 | 430.7 |
| Std. Deviation | 258.4 | 246.4 | 144.3 | 62.07 |
| Std. Error of Mean | 69.07 | 82.13 | 43.52 | 18.72 |
| Rheobase | | | |  |
| Descriptive statistics | *Dbx1*-lineage | *Dbx1*-lineage | *Foxp2*-lineage | *Foxp2*-lineage |
| Number of cells | 14 | 9 | 1 1 | 1 1 |
| Mean | 30.77 | 35.56 | 31 .82 | 31 .82 |
| Std. Deviation | 13.82 | 20.68 | 13.28 | 9.816 |
| Std. Error of Mean | 3.833 | 6.894 | 4.004 | 2.96 |
| Capacitance (pF) | | | |  |
| Descriptive statistics | *Dbx1*-lineage | *Dbx1*-lineage | *Foxp2*-lineage | *Foxp2*-lineage |
| Number of cells | 14 | 9 | 1 1 | 1 1 |
| Mean | 57.94 | 56.26 | 132.5 | 1 1 1 .7 |
| Std. Deviation | 33.39 | 23.75 | 94.55 | 58.49 |
| Std. Error of Mean | 8.923 | 7.915 | 28.51 | 17.64 |
